# High concentrations of atmospheric ammonia induce alterations of gene expression in the breast muscle of broilers (*Gallus gallus*) based on RNA-Seq

**DOI:** 10.1186/s12864-016-2961-2

**Published:** 2016-08-11

**Authors:** Bao Yi, Liang Chen, Renna Sa, Ruqing Zhong, Huan Xing, Hongfu Zhang

**Affiliations:** State Key Laboratory of Animal Nutrition, Institute of Animal Sciences, Chinese Academy of Agricultural Sciences, Beijing, 100193 China

**Keywords:** RNA-Seq, Breast muscle, Ammonia, Meat quality, Broiler (*Gallus gallus*)

## Abstract

**Background:**

High concentrations of atmospheric ammonia are one of the key environmental stressors affecting broiler production performance, which causes remarkable economic losses as well as potential welfare problems of the broiler industry. Previous reports demonstrated that high levels of ammonia altered body fat distribution and meat quality of broilers. However, the molecular mechanisms and metabolic pathways in breast muscle altered by high concentrations of ambient ammonia exposure on broilers are still unknown.

**Results:**

This study utilized RNA-Seq to compare the transcriptomes of breast muscles to identify differentially enriched genes in broilers exposed to high and low concentrations of atmospheric ammonia. A total of 267 promising candidate genes were identified by differential expression analysis, among which 67 genes were up-regulated and 189 genes were down-regulated. Bioinformatics analysis suggested that the up and down-regulation of these genes were involved in the following two categories of cellular pathways and metabolisms: Steroid biosynthesis (gga00100) and peroxisome proliferator-activated receptor (PPAR) signaling pathway (gga03320), which both participated in the lipid metabolism processes.

**Conclusions:**

This study suggests that longtime exposure to high concentrations of aerial ammonia can change fat content in breast muscle, meat quality and palatability via altering expression level of genes participating in important lipid metabolism pathways. These findings have provided novel insights into our understanding of molecular mechanisms of breast muscles exposed to ammonia in broilers. This study provides new information that could be used for genetic breeding and nutritional intervention in production practice of broilers industry in the future.

**Electronic supplementary material:**

The online version of this article (doi:10.1186/s12864-016-2961-2) contains supplementary material, which is available to authorized users.

## Background

Ammonia (NH_3_) is a colorless, highly irritant, alkaline gas which is produced during the decomposition of organic matter by bacterial deamination or reduction of nitrogenous substances [1, 2]. With the development of intensive large-scale poultry industry, air quality in housing is particularly poor [1, 3–5]. Ammonia as a major aerial pollutant of poultry buildings is causing increasing problems with broiler health and production.

Based on human and livestock safety, the currently recommended exposure limit for ammonia is set at 25 ppm [1, 6]. However, the levels of ammonia in broiler houses usually exceed 25 ppm, and even go high up to 80 ppm, when poultry are confined in buildings provided with artificial heat and ventilation (especially in cold winter of northern China) [3]. High concentrations of gaseous ammonia can have adverse effects on production performance [7]. The greatest problem caused by atmospheric ammonia is reduced growth performance, which results in lower body weight gain and higher feed conversion ratios. Ammonia toxicity was reported one century ago by Hahn et al. [8]. To date, most studies of ammonia toxicity have focused on the respiratory tract and nervous system in mammals [9–11]. Few detailed studies have been conducted on the breast muscle tissue response to ammonia exposure in poultry.

Exposure to high levels of ambient ammonia is known to have a detrimental effect on broiler production efficiency and meat yields. It has been shown that high concentrations of ammonia (70 ppm) can reduce growth performance and meat quality of broilers [12, 13]. Previous studies in our laboratory have shown that high concentrations of environmental ammonia can alter body fat distribution and lipid metabolism. Among body fat deposition related tissues of broilers, we focus on breast muscles which are the main source of chicken meat. Chicken meat is one of the most popular food commodities in the world and the second most preferred meat by Chinese consumers [14, 15]. However, few detailed studies have been conducted on the ammonia exposed breast muscle response in poultry and the molecular mechanisms underlying these effects are yet to be investigated.

Based on previous research, we hypothesized that exposure to high concentrations of atmospheric ammonia can confer negative effects on the muscle tissue of broilers via different mechanisms, which requires further study to elucidate. Previously, broiler chickens with different growth performance or fat content were compared using microarrays [16]. With the rapid development of systems-biology approach, high-throughput transcriptome sequencing can be used to discover genes that are functionally active and participate in specific biological processes [17–20]. In particular, Illumina sequencing technology can be used for gene identification with confirmed reliability [21, 22].

Currently, little is known about the alteration of genes in the breast muscle of poultry that have been exposed to high levels of atmospheric ammonia. Therefore, the objectives of the study reported herein were to utilize a transcriptome sequencing procedure to identify differential expressed genes in breast muscle tissues of broilers exposed to high and low concentrations of atmospheric ammonia. In conclusion, our results provide important information towards understanding the biological basis of variation in ammonia exposed broilers, also provide information towards comprehensively understanding the molecular mechanisms and metabolic pathway alteration of the physiological responses to ammonia which are critical to improve poultry production efficiency and welfare.

## Methods

### Ethics statement

This study was undertaken in strict accordance with the Regulations for the Administration of Affairs Concerning Experimental Animals of the State Council of the People’s Republic of China. The protocol was approved by the Committee on Experimental Animal Management of the Chinese Academy of Agricultural Sciences. Before tissue sampling, birds were humanely sacrificed by cervical dislocation. All efforts were made to minimize distress.

### Animals and exposure conditions

Ninety-six 1-day-old Arbor Acres (AA) male broilers were purchased from a commercial hatchery in Beijing (Beijing Arbor Acers Broiler Co., Beijing, China). All birds were housed in individual wire-bottom cages in an environmentally controlled room under standard brooding practices, and given *ad libitum* access to water and a maize-soybean basal diet during the first 21 days. Then, broilers were divided into two environmentally controlled exposure chambers. Each exposure chamber was a 4500 × 3000 × 2500 mm (length × width × height) sealed unit, sectioned for housing 48 birds per chamber. The diet during the experiment was formulated to achieve the National Research Council (NRC, 1994) recommended requirements for all nutrients containing ME, 12.77 MJ kg^-1^, and crude protein 19.93 % (Additional file 1: Table S1). The concentrated ammonia was delivered in a whole body animal exposure chamber from days 22 to 42, and the chambers were computer programmed to have the ammonia concentration as required. Temperature and airflow were controlled during the exposures to ensure adequate ventilation, minimize buildup of animal-generated contaminants (dander, CO_2_, H_2_S) and to avoid thermal stress [23, 24]. Broilers in the treatment group were exposed to 75 ± 3 ppm (parts per million) ammonia during the experimental period. Broilers in the control group were raised in a separate chamber with ambient ammonia concentration at 3 ± 3 ppm. The concentration of ammonia in both chambers was monitored with a LumaSense Photoacoustic Field Gas-Monitor INNOVA 1412 (Santa Clara, CA, USA) during the entire experiment. The experiment was carried out at the State Key Laboratory of Animal Nutrition in Beijing, China.

### Sample collection

At day 42, all birds were weighed after a 12 h-fasting (12 h food withdrawal) period. Sixteen birds (8 broilers each group) were randomly selected for breast muscle sample collection. The chickens were euthanized by manual cervical dislocation and then exsanguinated for tissue sampling. Samples of breast muscle were collected and prepared for carcass traits measurements. Carcass traits were determined in accordance with the method described by Połtowicz and Doktor [25]. Breast muscle was quickly dissected and weighed, then rapidly frozen in liquid nitrogen, and stored at −80 °C for further transcriptome (RNA-Seq) and qRT-PCR analyses.

### Total RNA extraction and cDNA library construction

The breast muscle of the 6 randomly selected broilers (3 birds per group) were separately ground in frozen state in liquid nitrogen. Total RNA of breast muscle was isolated with TRIzol reagent (Invitrogen) according to the manufacturer’s instructions. The concentration of RNA samples was measured using the NanoDrop 2000 (Nanodrop Technologies, Wilmington, DE). Agilent 2100 Bioanalyzer (Agilent Technologies, Santa Clara, CA) was utilized to assess the integrity of the total RNA (RIN number > 8.0). The total RNA with lowest quality was not used for further study.

A total amount of 5 μg RNA per sample was used as input material for the mRNA preparations. Briefly, mRNA was extracted from total RNA using oligo (dT) magnetic beads (Invitrogen) and sheared into short fragments of about 200 bases. These fragmented mRNAs were then used as templates for cDNA synthesis. The cDNAs were then PCR amplified to complete the library. After PCR enrichment, cDNA quantity and quality were assessed using a NanoDrop 2000 spectrophotometer (Nanodrop Technologies, Wilmington, DE) and Agilent 2100 Bioanalyzer (Agilent Technologies, Santa Clara, CA). After construction, the 6 cDNA libraries were normalized, as suggested by the manufacturer, to 10 nmol μl^−1^ using Tris buffer (10 mmol Tris–HCl, 0.1 % Tween 20, pH 8.5). Sequencing libraries were generated using the TruSeq RNA Sample Prep kit v2 (Illumina, San Diego, CA) following manufacturer’s instructions. The cDNA library was paired-end sequenced using an Illumina HiSeq 2000 platform. The resultant data was deposited in NCBI’s Gene Expression Omnibus (GEO) database (GEO accession: GSE84099).

### Quality control and primary analysis of reads

Before read alignment, the quality of raw sequence reads was checked using the FastQC program (http://www.bioinformatics.babraham.ac.uk/projects/fastqc/), clean reads (valid data) were obtained by removing low quality reads (threshold quality, 20; threshold length, 50 bp) as well as reads containing adapter sequences, ploy-N and the sequencing primer from the raw data. At the same time, Q30, GC-content and sequence duplication level of the clean data were calculated. The raw data generated from the Illumina Hiseq platform were filtered using a FastQC quality control analysis. All of the downstream analyses were based on high quality clean reads.

### Mapping reads to the chicken reference genome

Sequencing reads from each sample were mapped to the chicken reference genome [Ensembl Galgal4 (GCA_000002315.2)] using the TopHat program. Parameters of TopHat were set to allow only unique alignment to the reference genome. Reads with more than two mismatches were discarded, and concordant mapping for both reads in a pair was required. To obtain the mapping statistics, the alignment BAM files were further examined using RNA-SeQC. Reads were then annotated to reference genes using Bowtie2/Tophat2 software. The differentially expressed genes (DEGs) between control and treatment groups were identified using DESeq software (http://www-huber.embl.de/users/anders/DESeq/). Genes with a *P*-value of less than 5 % (i.e., pval < 0.05) and fold-change ≥ 2 were considered significant.

### Differential gene expression and functional analysis

The DEGs were classified for the categories of molecular functions, cellular component and biological process using gene ontology (GO) annotation. The Kyoto Encyclopedia of Genes and Genomes (KEGG) predicted the metabolic pathways of the DEGs. To identify potential common pathways, DEGs were analyzed for biological process and pathway enrichment using DAVID. Pathway enrichment analysis identifies significantly enriched metabolic pathways or signal transduction pathways using the corrected *P*-value < 0.05 as a threshold of significance.

### Confirmation of differential gene expression levels by quantitative real-time PCR (qRT-PCR)

To validate the repeatability and reproducibility of gene expression data obtained by RNA-Seq in breast muscle of broilers, we performed qRT-PCR on 15 randomly selected genes. Total RNA was isolated by TRIzol reagent (Invitrogen). The first-strand cDNA was synthesized with M-MLV (Promega). Gene specific primers were designed according to the gene sequence using primer premier 5.0, which were synthesized by Sangon Biotech (Shanghai, China) (Additional file 2: Table S2). qRT-PCR was performed using a SYBR Fast qPCR Master Mix (Takara). The reaction mixtures were incubated in a 96-well plate at 95 °C for 20 s, followed by 40 cycles of 95 °C for 3 s and 60 °C for 30 s. All measurements were conducted in triplicates. The chicken glyceraldehyde-3-phosphate dehydrogenase (*GAPDH*) gene was used as an internal control. The 2^-ΔΔCt^ method was used to analyze relative RNA expression.

### Statistical analysis

Gene functions and related gene ontology (GO) terms for relevant gene lists were generated using the DAVID bioinformatics database (https://david.ncifcrf.gov/) [26]. The heat map was generated using R package “gplots” [27], MA and volcano plots were generated using R package “ggplot2”. Data on gene expressions were analyzed by one-way ANOVA (SAS Version 9.2, SAS institute Inc., Cary, NC) and JMP 10 software (SAS Inst. Inc., Cary, NC). A group difference was assumed to be statistically significant when *P* < 0.05. All results were expressed as mean ± S.D.

## Results

### Effects of high concentrations of atmospheric ammonia on growth performance and body fat distribution in broilers

In this study, experiments for all birds (control and treatment groups) began on day 22–42. During the entire experimental period (21 days), birds exposed to ammonia had less average daily gain (ADG) (*P* < 0.05) and less average daily feed intake (ADFI) (*P* < 0.05). The feed conversion ratio (FCR) for the treatment group was greatly increased (*P* < 0.05) compared with the control group (Table 1). Ammonia exposure altered body fat distribution of broiler chickens, fat in liver and breast muscle decreased (*P* < 0.05), however, abdominal fat increased significantly (*P* < 0.05) (Table 1).

**Table 1:** Effects of atmospheric ammonia on growth performance and body fat distribution in broiler chickens

	Groups	
	Control	Treatment
ADG (g/d)	99.41 ± 10.08^a^	67.92 ± 12.68^b^
ADFI (g/d)	158.42 ± 5.78^a^	125.56 ± 1.50^b^
FCR (g feed/g weight gain)	1.64 ± 0.18^b^	1.80 ± 0.25^a^
Breast muscle (%)	23.61 ± 1.06	22.09 ± 2.28
Fat in breast muscle (%)	1.14 ± 0.18^a^	0.78 ± 0.14^b^
Liver (%)	19.53 ± 1.60	19.32 ± 1.90
Fat in liver (%)	5.41 ± 1.18^a^	3.09 ± 0.17^b^
Abdominal fat (%)	1.62 ± 0.10^b^	2.05 ± 0.45^a^

Values within a row not sharing a common superscript letter indicate significant difference between groups at *P* < 0.05. Numbers are mean ± S.D

*ADG* average daily gain, *ADFI* average daily feed intake, *FCR* feed conversion ratio

^a, b^ within a row indicate significant difference between groups at *P* <0.05

### Overall assessment for mapping statistics

**Fig. 1:**
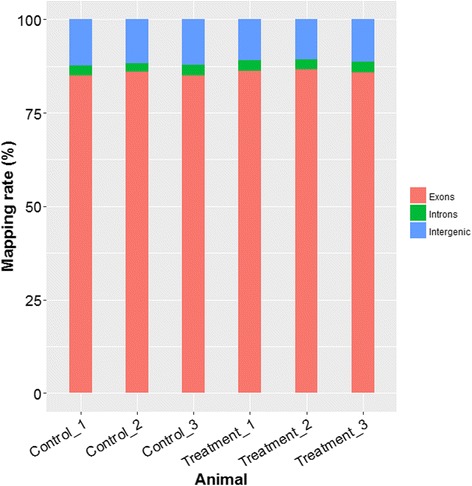
The percentage of reads mapped to exonic, intronic and intergenic regions

The RNA-Seq of six breast muscle samples yielded around 474.5 million of raw paired-end reads. After quality filtering, each sample remained approximately 7.5 gigabases (Gb) high-quality sequence data, ranging from 6.7 to 9.5 Gb. Using TopHat2 software, more than 70.36 % of clean reads per sample were mapped back to the reference genome after alignment. Almost 65.37–68.23 % reads were aligned in a unique manner, while 4.72–5.54 % as multiple-mapped reads. The summary of alignment for all samples is shown in Table 2. Among total mapped reads, the vast majority of which (85.01–86.66 %) fell into annotated exons, 10.64–12.31 % was within the large intergenic territory, and only 2.33–2.90 % was located in introns (Fig. 1).

**Table 2:** Summary statistics for sequence quality and alignment information of six breast muscle samples in two groups

Sample	C_1	C_2	C_3	T_1	T_2	T_3
Group	Control	Control	Control	Treatment	Treatment	Treatment
Raw reads	73,139,016	73,988,890	70,579,138	74,086,562	82,677,190	100,025,614
Clean reads	69,375,390	70,089,536	66,879,214	70,162,270	78,368,448	94,553,606
Valid ratio (%)	94.81 %	94.68 %	94.71 %	94.66 %	94.75 %	94.49 %
Q30 (%)	94.90 %	94.75 %	94.79 %	95.04 %	95.11 %	94.92 %
GC content (%)	51.50 %	51.50 %	51.50 %	51.50 %	51.50 %	51.50 %
Total mapped reads	49,236,780	49,313,882	47,427,718	50,794,372	57,048,473	69,576,797
Uniquely mapped reads	45,869,880	45,818,693	44,268,019	47,090,588	52,708,435	64,514,418
Multiple mapped reads	3,366,900	3,495,189	3,159,699	3,703,784	4,340,038	5,062,379
Mapping rate (%)	70.97 %	70.36 %	70.92 %	72.40 %	72.80 %	73.58 %

The relative expression of a gene is normalized as fragments per kilobase of transcript per million mapped fragments (FPKM), which is proportional to the number of cDNA fragments originated from the gene transcript. The lowest limit of gene expression value is set to be 0.5 FPKM in at least one of the 6 samples. According to this limit, 11,340 genes are identified as being expressed in the breast muscle tissues (Additional file 3: Table S3). The correlation analysis based on the gene expression profiles found that the correlations among 3 samples were greater than 0.92 (Additional file 4: Figure S1).

### Gene differential expression analysis

**Fig. 2:**
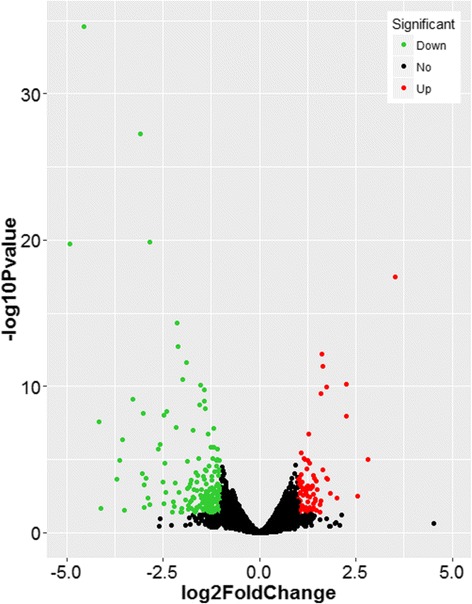
Volcano plot. Red dots (Up) represent significantly up-regulated genes (*P* < 0.05, fold change ≥ 2); green dots (Down) represent significantly down-regulated genes (*P* < 0.05, fold change ≤ 0.5); black dots (No) represent insignificantly differential expressed genes

**Fig. 3:**
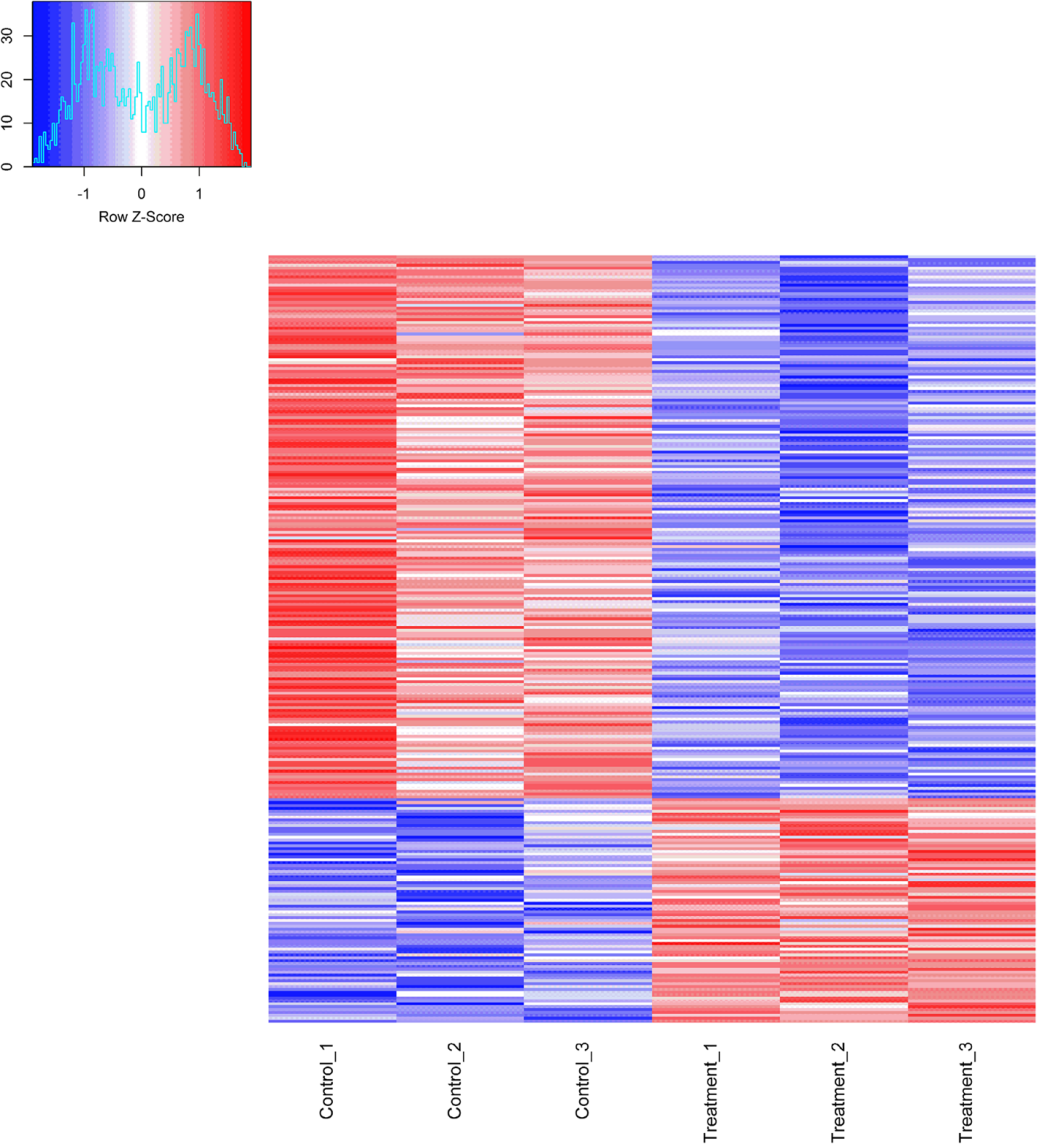
The relative expression level (in FPKM) of differentially expressed genes between control and treatment groups. The X-axis is sample expression pattern in different concentrations of ammonia exposed groups. The first three samples are in control group; the latter three samples are in treatment group. The Y-axis is the gene clusters across groups

DESeq software was used for subsequent differential analysis. In total, 267 genes were differentially expressed at a fold-change ≥ a space 2 or ≤ 0.5 at *P* < 0.05 in response to high level ammonia exposure (Additional file 5: Table S4 and Fig. 2). Of these genes, 189 genes were down-regulated and 78 were up-regulated (Fig. 2 and Additional file 6: Figure S2). The top ten up and down-regulated genes are listed in Table 3. The fold changes induced by high ammonia ranged from −30.4 to 11.4 (Fig. 3 and Table 3).

**Table 3:** Top 10 down- and up-regulated genes in treatment group compared to control group

Gene ID	Gene symbol	Fold change
Down-regulated genes		Down
NM_001199909.1	*PDK4*	30.4
XM_004940680.1	*FHL1*	23.5
NM_001277411.1	*CA3*	18.1
NM_001167752.2	*MB*	17.3
XM_004937471.1	*PFKFB3*	13.0
XM_004937841.1	*MYBPC1* ^*a*^	12.4
XM_416332.4	*MYBPC1* ^*b*^	11.8
NM_001005431.1	*FKBP5*	11.4
NM_001030956.1	*FBXO32*	9.8
XM_416965.4	*METTL21C*	8.5
Up-regulated genes		Up
XM_004950318.1	*LOC101748351*	11.4
XM_419014.3	*SBK2*	7.0
XM_004938015.1	*CYB5R3*	5.8
XM_417174.4	*DCUN1D5*	4.8
NM_204672.1	*SNCG*	4.8
NM_001007477.3	*LOC396531*	4.0
XM_004944616.1	*LOC770936*	3.6
XM_419574.4	*C1ORF96*	3.4
NM_001001766.1	*LIMS1*	3.4
XM_004946388.1	*DNAJC30*	3.4

*PDK4*, pyruvate dehydrogenase kinase, isozyme 4, *FHL1* four and a half LIM domains 1 *CA3*, carbonic anhydrase III, muscle specific, *MB* myoglobin, *PFKFB3* 6-phosphofructo-2-kinase/fructose-2,6-biphosphatase 3, *MYBPC1* ^a^ myosin binding protein C, slow type, transcript variant X1, *MYBPC1* ^b^ myosin binding protein C, slow type, transcript variant *X*2, *FKBP5* FK506 binding protein 5, *FBXO32* F-box protein 32, *METTL21C* methyltransferase like 21C, *LOC101748351* spindle and kinetochore-associated protein 1-like, *SBK2* SH3-binding domain kinase family, member 2, *CYB5R3* cytochrome b5 reductase 3, *DCUN1D5* DCN1, defective in cullin neddylation 1, domain containing 5, *SNCG* synuclein, gamma (breast cancer-specific protein 1), *LOC396531* parvalbumin, *LOC770936* serine/threonine-protein kinase SRPK3-like, *C1ORF96* chromosome 3 open reading frame, human C1orf96, *LIMS1* LIM and senescent cell antigen-like domains 1, *DNAJC30* DnaJ (Hsp40) homolog, subfamily C, member 30

### Confirmation of RNA-Seq experiment

**Fig. 4:**
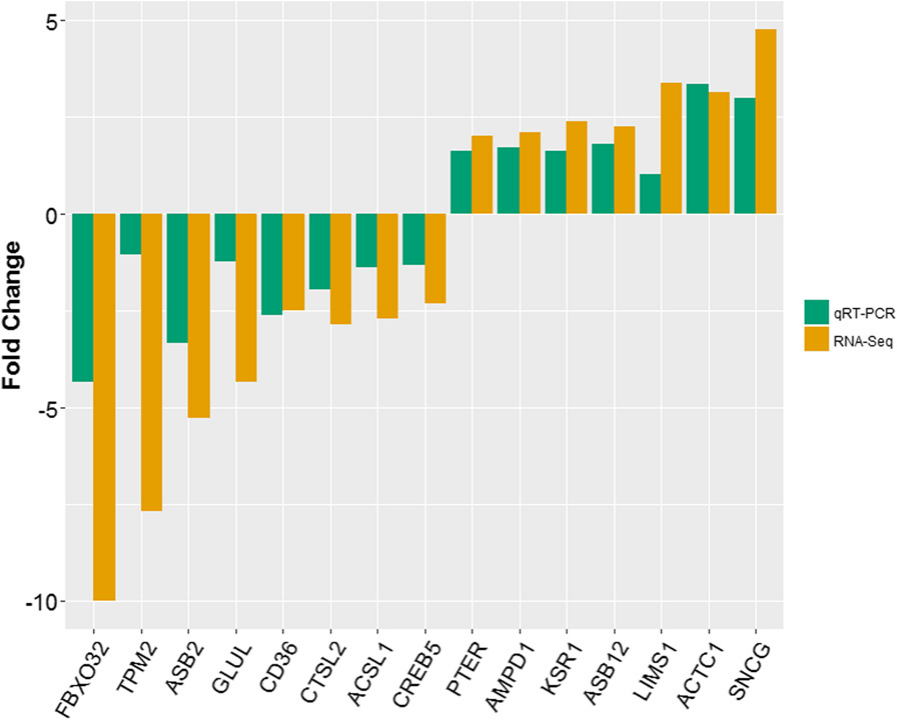
qRT-PCR validation of differential expressed genes from breast muscle of 42-day-old AA broilers. *GAPDH* was used as an internal control, and data are presented as fold change (*n* = 3 per group)

To evaluate the reliability of results from RNA-Seq, qPCR was performed using aliquots of non-pooled RNA samples. Fifteen protein-coding genes (*FBXO32, TPM2, ASB2, GLUL, CTSL2, ACSL1, CD36, CREB5, PTER, AMPD1, ASB12, KSR1, ACTC1, LIMS1, SNCG*) were randomly selected. The results showed a high consistency between RNA sequencing and the qPCR methods (Fig. 4), and confirmed that the relative gene expression of RNA-Seq was reliable.

### GO annotations and pathway analysis of differential expressed genes

**Fig. 5:**
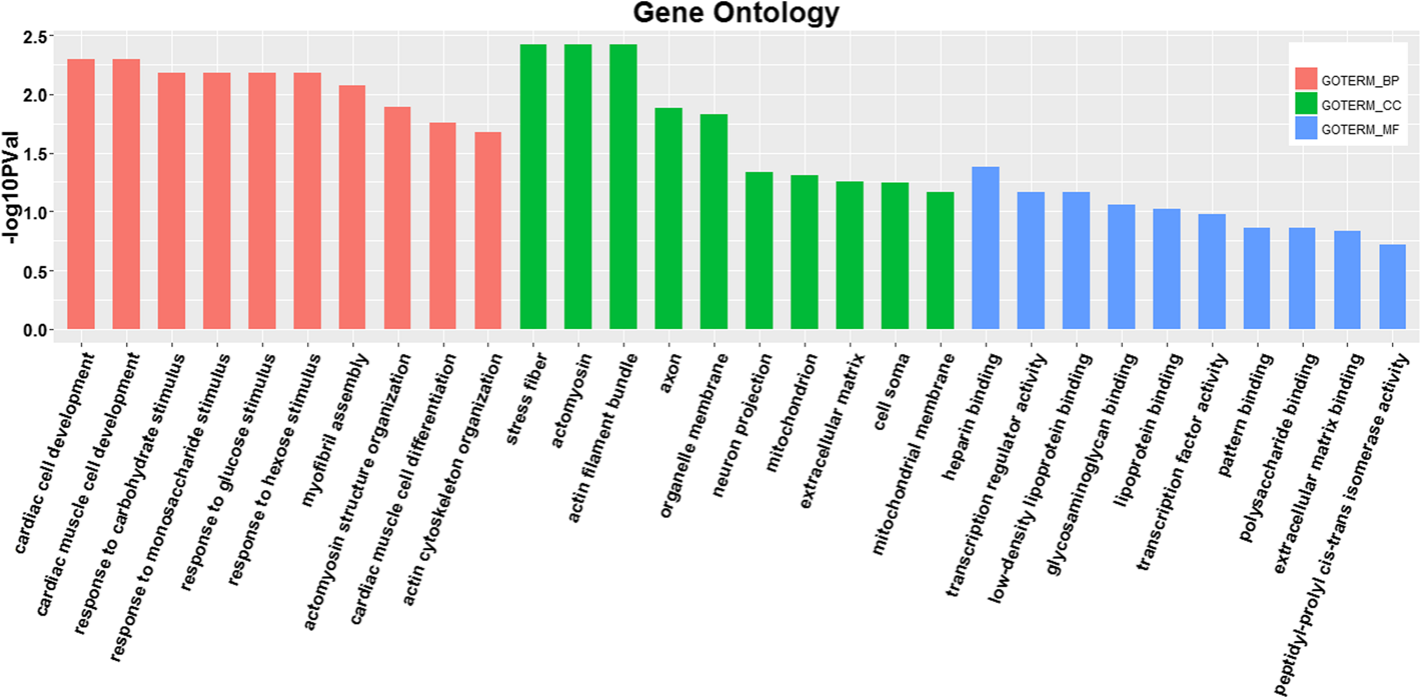
Gene ontology (GO) annotation of differentially expressed genes. GOTERM_BP, biological process; GOTERM_CC, cellular component; GOTERM_MF, molecular function

**Fig. 6:**
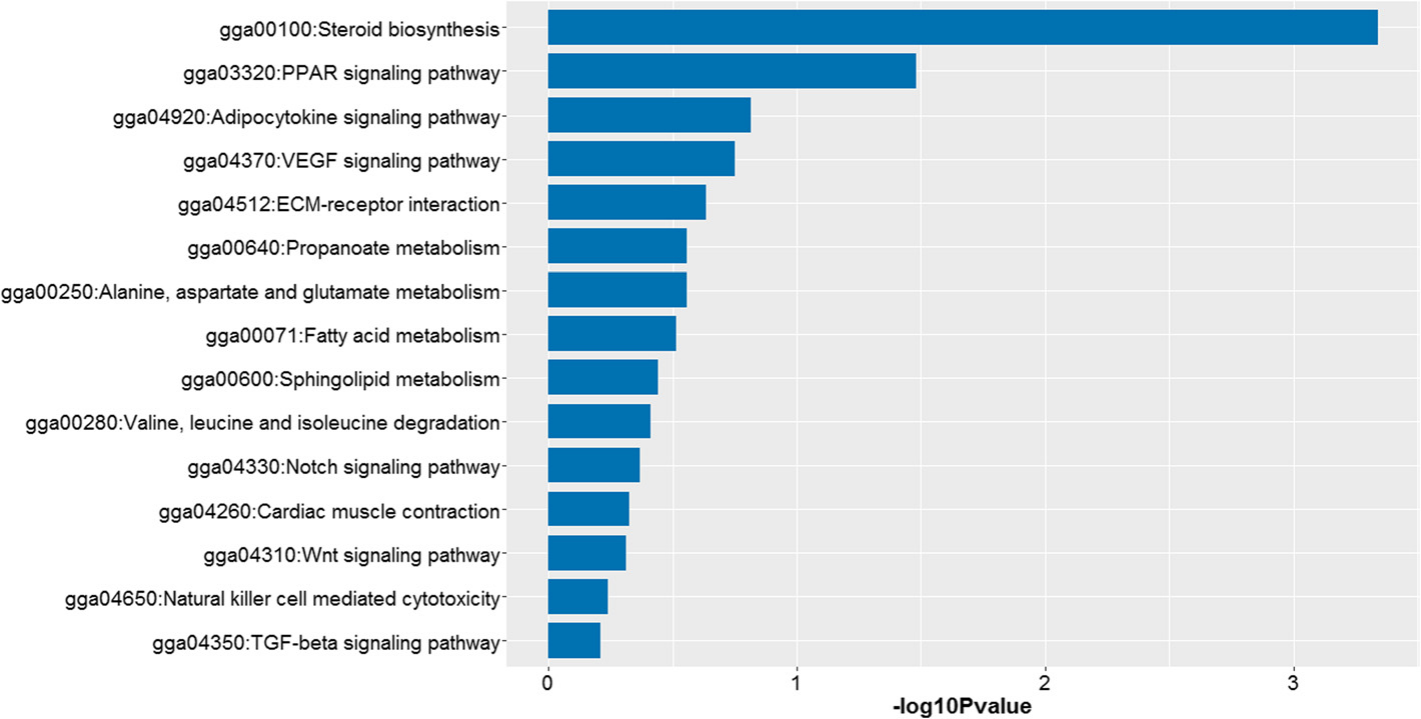
KEGG Pathway analysis of differentially expressed genes

Gene ontology (GO) was used to evaluate the function of differentially expressed genes in two groups. All the differentially expressed genes were performed by gene ontology terms through the DAVID platform (Fig. 5). In the biological process category, the genes that participate in cellular development, response to stimulus (carbohydrate and monosaccharide) were at the top ratio in the differentially expressed genes, in the cellular component group, the differentially expressed genes are concentrated in “stress fiber” and “actomyosin” (Fig. 5). The main molecular functional group of differentially expressed genes are related to binding (heparin, low-density lipoprotein and lipoprotein binding) and transcription regulator activity. The KEGG pathway analysis revealed two overrepresented pathways, including steroid biosynthesis and peroxisome proliferator-activated receptor (PPAR) signaling pathway (Fig. 6 and Table 4).

**Table 4:** Significantly enriched pathways for differential expressed genes

Pathway name	Enriched genes	*P*-value
Steroid biosynthesis	*DHCR24*, *DHCR7*, *HSD17B7*, *LSS*	0.00
PPAR signaling pathway	*ACSL1*, *CPT-1A*, *CD36*, *SLC27A1*	0.03

*DHCR24* 24-dehydrocholesterol reductase, *DHCR7* 7-dehydrocholesterol reductase, *HSD17B7* hydroxysteroid (17-beta) dehydrogenase 7, *LSS* lanosterol synthase (2,3-oxidosqualene-lanosterol cyclase), *ACSL1* acyl-CoA synthetase long-chain family member, *CPT-1A* carnitine palmitoyltransferase 1A (liver), *CD36* CD36 molecule (thrombospondin receptor), *SLC27A1* solute carrier family 27 (fatty acid transporter), member 1

## Discussion

Breast muscle tissue is important in broilers, which is the main source of chicken meat [28, 29]. Alterations in muscles metabolism pathways induced by longtime ammonia exposure may lead to the decline of meat yields and eating quality [12, 23, 30, 31]. In the present study, high-throughput transcriptomic analysis was performed to elucidate the molecular mechanisms in broiler muscles exposed to high and low concentrations of aerial ammonia. Consistent with previous observations, our data showed a negative correlation between ammonia exposure and growth performance in birds [1, 3, 7, 24].

The results obtained in our study indicated that analyzed control and treatment groups differed in the expression of 267 transcripts (*P* < 0.05). As fat content in breast muscles decreased, consistent with our expectations, these differential expressed genes major participated in two lipid metabolism-related pathways (gga00100 and gga03320). Differential expressed genes involved in PPAR signaling pathways here have been proven to be functional in lipid metabolism and fat deposition including *CD36* (CD36 molecule), *SLC27A1* (solute carrier family 27, member 1), and *ACSL1* (acyl-CoA synthetase long-chain family member 1). The above mentioned differential expressed genes in lipid metabolism may contribute to the lower fat deposition in muscles.

It has been reported that both the *FAT/CD36* and *ACSL* facilitate the transport of fatty acids released by lipoprotein lipase [32]. *CD36*, localizing to the plasma membrane and mitochondria as a fatty acids receptor, functions in fatty acids uptake, transport and oxidation [33, 34], and is shown to be highly expressed in skeletal muscle tissues that require energy of fatty acids oxidation [35]. McFarlan et al. found that *CD36* knockout mice hindered fatty acids transport, oxidation, and decreased lipid content in muscles [36]. Consistent with that, in the current study, *CD36* was 1.5 times lower in high level ammonia exposed group than control group, resulting in lower fat deposition in muscles. Joseph et al. have shown that *ACSL1* participates in the biosynthesis of unsaturated fatty acids and fatty acid metabolism, playing a key role in lipid metabolism [37]. In pig breeds, *ACSL1* gene might be related to the capacity of fat deposition and meat quality [38]. Therefore, *ACSL1* may function an analogous role in broilers in our ammonia exposure experiment. *SLC27A1* is another key gene involved in fatty acids transport and lipid metabolism. It is mostly expressed at muscle and adipose tissues, Wu et al. have found that inactivation of *SLC27A1* can protect mouse from diet-induced obesity [39, 40]. High level ammonia exposure decreased the expression of *SLC27A1* leading to negative effects to adipogenesis in breast muscles may through similar mechanisms provided by Wu et al. In this study, a high level of ammonia exposure decreased the expression of *CD36*, *ACSL1* and *SLC27A1* blocking fat deposition, which is in agreement with the results of above mentioned research.

Lipid metabolism is important in muscle tissue of broilers, which is correlated with meat quality parameters and palatability of consumers [41, 42]. Moreover, high concentrations of aerial ammonia had a detrimental effect on meat tenderness and eating quality [12, 23]. Tenderness is a determinant parameter of meat quality labeled by shear force, Sackett et al. have showed that broilers exposed to 75 ppm ammonia significantly increased shear values in breast muscle, decreased meat tenderness and quality [43]. Taken together, high concentrations of ambient ammonia exposure can cause the decline of tenderness in breast muscle, and lead to decrease of chicken meat quality [12, 44–46]. Previous reports have shown that the gene *ASB2* (Ankyrin repeat and SOCS Box containing 2) possess higher expression levels in a high tenderness broiler group than in a low tenderness of chicken group in breast muscles [13, 47], suggesting that it may participate in the tenderization process in broiler chickens. *ASB2* was regarded as a participant in the tenderization process in broiler chickens as its product results in the degradation of filamins, proteins which form muscle fibres [12, 48, 49]. In our study, a high level of ammonia exposure decreased the expression of *ASB2* which was also confirmed by qRT-PCR.

Besides, in the current research, another differential expressed gene characterized by negative effects on breast meat quality in broilers was also observed. The *PLIN2* (perilipin 2) gene has been reported to be involved in the regulation of intracellular lipid storage and fatty mobilization [50]. Conte et al. showed that the high expression of *PLIN2* is associated with decreased muscle strength [51]. In pigs, increased *PLIN2* mRNA expression was detected in the skeletal muscle of pigs with higher intramuscular fat content [52–54]. As we all know, tenderness and intramuscular fat content both are important parameters of meat eating quality [55–58]. Taken together, we proposed that, in this study, *PLIN2* gene expression abundance altered by ammonia may impair meat quality by increasing shear force meanwhile decreasing fat content in muscles.

In summary, the breast muscle transcriptome analysis demonstrated that broilers exposed to high concentrations of atmospheric ammonia altered body fat content and meat quality through changing the expression abundance of key lipid metabolism genes (such as *CD36*, *ACSL1*, *SLC27A1, ASB2 and PLIN2*) and pathways (gga00100 and gga03320).

## Conclusions

In conclusion, high levels of ammonia resulting in fat content changes in breast muscle tissues can be characterized through high-throughput RNA sequencing analysis. The present transcriptome analysis has demonstrated that exposure to high concentrations of aerial ammonia leads to differential abundances of a number of genes (*CD36*, *ASB2*, *ACSL1, PLIN2* and etc*.*) in breast muscles of broilers. To our knowledge, this is the first study of the relationships between gene expression in breast muscle tissues and aerial ammonia exposure in broiler chickens. The ammonia may play a negative role in meat quality by decreasing the lipid content, but the detailed molecular mechanisms of lipid deposition altered by ammonia still need to be further elucidated (perhaps varying lengths of ammonia exposure). The results of our study provide a foundation for future investigations into the gene interactive networks involved in the response to ammonia, and for the development of strategies to avoid the negative impact of ammonia on animal productivity in broiler industry. This study provides theory basis for environmental control (ammonia) and animal welfare in poultry industry.

## Abbreviations

AA, arbor acres; ADFI, average daily feed intake; ADG, average daily gain; PPAR, peroxisome proliferator-activated receptor; DEGs, differentially expressed genes; FCR, feed conversion ratio; FPKM, fragments per kilobase of transcript per million mapped fragments; *GAPDH*, glyceraldehyde-3-phosphate dehydrogenase; GO, gene ontology; KEGG, Kyoto Encyclopedia of Genes and Genomes; NH_3_, ammonia; NRC, National Research Council

## Additional files

Additional file 1: Table S1. Composition of the experimental diet and calculated proximate composition of the diet. (DOCX 14 kb) Additional file 2: Table S2. The qPCR primers used for verification of the differentially expressed genes of the AA broiler breast muscle tissues. (DOCX 17 kb) Additional file 3: Table S3. Genes expressed in the breast muscle tissues of broilers are identified using sequencing. (XLSX 2831 kb) Additional file 4: Figure S1. The correlation analysis. (TIF 177 kb) Additional file 5: Table S4. Differentially expressed genes in chickens between control and treatment groups. (XLSX 41 kb) Additional file 6: Figure S2. MA plot. (TIFF 1966 kb)
