# Supplementary material for: High concentrations of atmospheric ammonia induce alterations of gene expression in the breast muscle of broilers (Gallus gallus) based on RNA-Seq
Source: BMC Genomics. 2016 Aug 11;17:598. doi: 10.1186/s12864-016-2961-2 (PMC4982197; doi:10.1186/s12864-016-2961-2)
Supplement: Additional file 1: Table S1. — Composition of the experimental diet and calculated proximate composition of the diet. (DOCX 14 kb) [file 12864_2016_2961_MOESM1_ESM.docx]

**Table S1 Composition of the experimental diet and calculated proximate composition of the diet.**

| Ingredients (%) g/kg dry weight | |
| --- | --- |
| Maize | 58 |
| Soybean meal | 33.4 |
| Soybean oil | 4 |
| Limestone | 1.15 |
| Calcium hydrophosphate | 1.65 |
| Lysine | 0.18 |
| Methionine | 0.32 |
| Choline chloride (50%) | 0.06 |
| Sodium chloride | 0.25 |
| Premix^*^ | 1 |
| Total | 100 |
| Calculated nutrient and energy levels | g/kg dry weight |
| Crude protein (%) | 19.93 |
| Calcium (%) | 0.09 |
| Available P (%) | 0.40 |
| Lysine (%) | 1.14 |
| Methionine (%) | 0.50 |
| ME (MJ/kg) | 3.05 |

^*^Providing the following (g/kg fresh weight), Vitamin A, 6,000 IU; Vitamin D: 1,000 IU; Vitamin E: 75.0 mg; Vitamin K_3_, 18.8 mg; Vitamin B_1_, 9.8 mg; Vitamin B_2_, 28.8 mg; Vitamin B_6_, 19.6 mg; Vitamin B_12_, 0.1 mg; calcium pantothenate, 58.8 mg; nicotinic acid, 196.0 mg; folic acid, 4.9 mg; biotin, 2.5 mg; Cu (copper sulfate), 4.0 mg; Fe (ferrous sulfate), 40.0 mg; Zn (zinc sulfate), 37.6 mg; Mn (manganese sulfate), 50.0 mg; Se (sodium selenite), 0.2 mg; I (potassium iodide), 0.2 mg. ME was a calculated value. Other nutrients levels were measured values.
