# Supplementary material for: High concentrations of atmospheric ammonia induce alterations of gene expression in the breast muscle of broilers (Gallus gallus) based on RNA-Seq
Source: BMC Genomics. 2016 Aug 11;17:598. doi: 10.1186/s12864-016-2961-2 (PMC4982197; doi:10.1186/s12864-016-2961-2)
Supplement: Additional file 2: Table S2. — The qPCR primers used for verification of the differentially expressed genes of the AA broiler breast muscle tissues. (DOCX 17 kb) [file 12864_2016_2961_MOESM2_ESM.docx]

**Table S2 The qPCR primers used for verification of the differentially expressed genes of the AA broiler breast muscle tissues**.

| **Gene symbol** | **Accession no.** | **Primer sequence** | **Product size (bp)** |
| --- | --- | --- | --- |
| *GAPDH* | NM_204305 | 5'-CACTGTCAAGGCTGAGAACGG-3'  5'-GAGATGATAACACGCTTAGCACCA-3' | 192 |
| *FBXO32* | NM_001030956 | 5'-ATTCGCAAACGGCTAATCCT-3'  5'-GCGTGTCACCATACTGCTCCTT-3' | 106 |
| *TPM2* | NM_205446.1 | 5'-TTTGCTGAGCGGTCTGTGGC  5'-GCTCCTCGCTGATGGCTTTGT | 100 |
| *ASB2* | XM_015287761 | 5'-CAACAGCCACAACCCTCCCT-3'  5'-TCCATCCCTGATTGCTGCTAC-3' | 178 |
| *GLUL* | NM_205493 | 5'-TGGAGGAACCAACGCAGAAGTG-3'  5'-ACCCGGTGGAGGATGAAACG-3' | 114 |
| *CTSL2* | NM_001168009 | 5'-GATGAAGACTGTCGCTACAAGG-3'  5'-ACACTGGACCCACAGATGCT-3' | 118 |
| *ACSL1* | NM_001012578 | 5'-AATACAGGCAAGTTTGGGAGG-3'  5'-ACCAGGCATTGACAGTGAGC-3' | 163 |
| *CD36* | NM_001030731 | 5'-TGGGAAGGTTACTGCGATTT-3'  5'-TCACGGTCTTACTGGTCTGGT-3' | 142 |
| *CREB5* | XM_004939382 | 5'-AGACAGCCTATGCCAGCCTCTATG-3'  5'-GAGTCAGTGCTGCCTTCAATCTCA-3' | 211 |
| *PTER* | XM_015281996 | 5'-CAGGAGATAGATGCCGTGAGG-3'  5'-TGTTTGAGAAGAATGCGTGGA-3' | 189 |
| *AMPD1* | XM_004935003 | 5'-TACCGTGCCTTGTGCATCCG-3'  5'-GCCAGCATCACTTGCCCTCC-3' | 120 |
| *ASB12* | NM_001277670 | 5'-CCCTCTGGCAGCATCTACAACA-3'  5'-GGTAAAGCGGACCGGAACAAG-3' | 166 |
| *KSR1* | XM_015295862 | 5'-GAGGCACCTGCTTGCAGAAT -3'  5'-AAGATGGCGTGGAAGAAGTTGTA-3' | 214 |
| *ACTC1* | NM_001079481 | 5'-TGGCAGGGATCTGACCGACTA-3'  5'-TCCAGGGAGGATGACGAAGC-3' | 168 |
| *LIMS1* | NM_001001766.1 | 5'-AAGCCATTCCTGGGTCATCG  5'-TCACGCACCACGCCTTATTC | 151 |
| *SNCG* | NM_204672 | 5'-GGCAGTGGTAGCCAGCGTGAA-3'  5'-GCGGAGCCAGGTCCTCCTTT-3' | 104 |

*GAPDH* = glyceraldehyde-3-phosphate dehydrogenase

*FBXO32* = F-box protein 32

*TPM2* = tropomyosin 2 (beta)

*ASB2* = ankyrin repeat and SOCS box containing 2

*GLUL* = glutamate-ammonia ligase

*CTSL2* = cathepsin L2

*ACSL1* = acyl-CoA synthetase long-chain family member 1

*CD36* = CD36 molecule (thrombospondin receptor)

*CREB5* = cAMP responsive element binding protein 5

*PTER* = phosphotriesterase related

*AMPD1* = adenosine monophosphate deaminase 1

*ASB12* = ankyrin repeat and SOCS box containing 12

*KSR1* = kinase suppressor of ras 1

*ACTC1* = actin, alpha, cardiac muscle 1

*LIMS1* = LIM and senescent cell antigen-like domains 1

*SNCG* = synuclein, gamma (breast cancer-specific protein 1)
