# Supplementary figures and images for: High concentrations of atmospheric ammonia induce alterations of gene expression in the breast muscle of broilers (Gallus gallus) based on RNA-Seq
Source: BMC Genomics. 2016 Aug 11;17:598. doi: 10.1186/s12864-016-2961-2 (PMC4982197; doi:10.1186/s12864-016-2961-2)

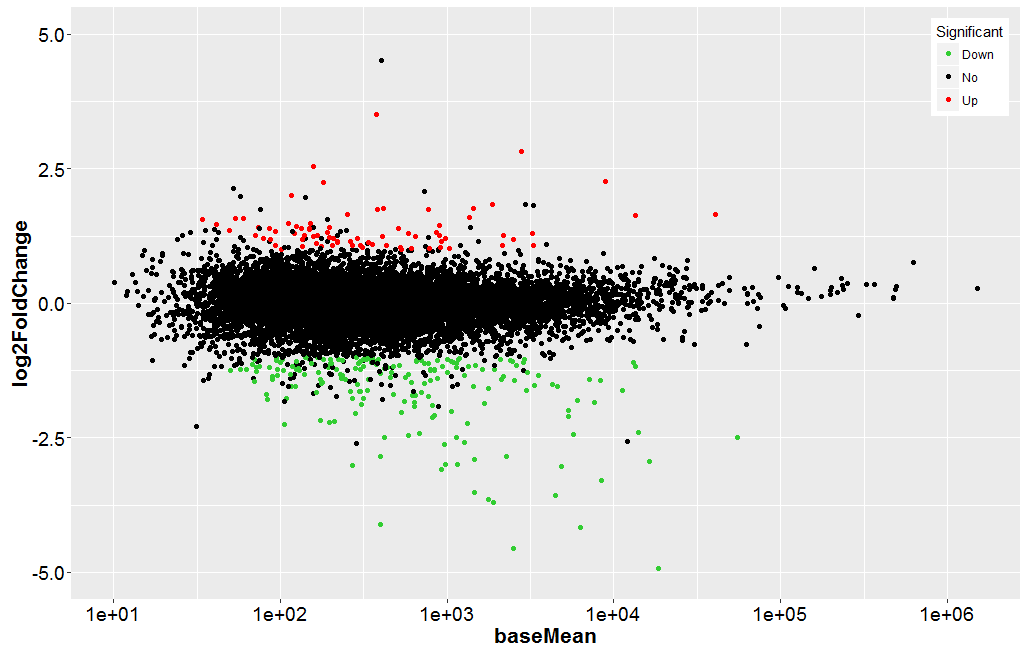

Supplement: Additional file 6: Figure S2. — MA plot. (TIFF 1966 kb) [file 12864_2016_2961_MOESM6_ESM.tiff]
